# Integrating skeletal muscle index and body roundness index for predicting functional outcomes in acute stroke patients: a prospective observational study

**DOI:** 10.3389/fneur.2025.1643247

**Published:** 2025-10-30

**Authors:** Haixia Huang, Yuanyuan Li, Haitao Zhang, Xinli Xiong

**Affiliations:** ^1^Department of Rehabilitation Medicine, Jiangwan Hospital of Hongkou District, Shanghai, China; ^2^Department of Neurology, Jiangwan Hospital of Hongkou District, Shanghai, China; ^3^Department of Radiology, Jiangwan Hospital of Hongkou District, Shanghai, China; ^4^Department of Neurology, The East Hospital Affiliated to Tongji University, Shanghai, China

**Keywords:** acute ischemic stroke, sarcopenia, skeletal muscle index (SMI), body roundness index (BRI), functional outcomes

## Abstract

**Background:**

After stroke, many patients experience dysphagia, anorexia, and metabolic stress, which may lead to malnutrition and accelerated loss of skeletal muscle mass. Sarcopenia and body fat distribution abnormalities significantly impact functional outcomes in acute stroke patients. While the skeletal muscle index (SMI) and body roundness index (BRI) have been studied individually, their combined predictive value for poor prognosis remains unclear. This study evaluates the integration of SMI and BRI for predicting unfavorable functional outcomes in acute stroke patients.

**Methods:**

A single-center, prospective cohort study was conducted on 123 acute ischemic stroke patients admitted within 3 days of onset. In this acute cohort, standardized strength/performance testing at admission was not feasible due to hemiparesis. Therefore, low skeletal muscle mass (L3-SMI) was used as the primary exposure. SMI was measured at the L3 vertebra using MRI, with sex-specific thresholds informed by EWGSOP2/AWGS muscle-quantity criteria. BRI was calculated based on waist circumference and height. Functional outcomes were assessed at 90 days post-discharge using the modified Rankin scale (mRS). Multivariate logistic regression and receiver operating characteristic (ROC) analyses were used to evaluate the independent and combined predictive abilities of SMI and BRI.

**Results:**

Patients with sarcopenia had significantly lower SMI (33.722 ± 3.307 cm^2^/m^2^) compared to non-sarcopenia patients (47.484 ± 5.934 cm^2^/m^2^, *p* < 0.001). Univariate analysis showed that lower SMI (OR = 0.90, 95% CI: 0.84–0.95, *p* < 0.001) and higher BRI (OR = 1.86, 95% CI: 1.21–2.85, *p* = 0.005) were associated with poor outcomes. Multivariate regression confirmed that sarcopenia (OR = 33.470, 95% CI: 7.118–157.394, *p* < 0.001) and BRI (OR = 2.200, 95% CI: 1.212–3.992, *p* = 0.010) independently predicted unfavorable outcomes. Combining SMI and BRI achieved an AUC of 0.933, demonstrating superior predictive performance compared to individual metrics. Decision curve analysis further highlighted the clinical utility of the combined model.

**Conclusion:**

The integration of SMI and BRI suggests a promising, hypothesis-generating model for identifying patients at risk of unfavorable outcomes after acute stroke, which may support early recognition and individualized management. These exploratory findings require further validation in larger, multicenter studies to confirm their robustness and generalizability.

## Introduction

Stroke is one of the most significant contributors to global mortality and disability (1). The early identification of risk factors is important for better prognosis and optimization of interventions in acute stroke patients. During the last decade, sarcopenia has been one of the most critical determinants of stroke outcomes (2–4). It is a complex geriatric syndrome featuring the loss of skeletal muscle mass and function (5). Studies have consistently demonstrated that sarcopenia in stroke patients is associated with poorer rehabilitation outcomes, delayed functional recovery, and higher risks of recurrent stroke due to possible mechanisms leading to heightened inflammation, metabolic dysregulation, and restricted physical activities (6–9).

However, not all stroke patients have sarcopenia. After stroke, many patients experience dietary disturbances such as dysphagia, anorexia, and increased catabolic stress, which can result in malnutrition and accelerate the decline of skeletal muscle mass. These nutrition-related changes may contribute to reduced muscle quantity (low SMI) and functional deterioration, even in the absence of clinically defined sarcopenia (6, 10, 11).

Skeletal muscle index (SMI) is the most well-known parameter of sarcopenia that defines the quantity of muscle loss objectively. It is measured as the cross-sectional area of the skeletal muscle at the level of the L3 vertebra through different imaging techniques (12). However, sarcopenia itself is not a critical issue in poor functional outcomes among stroke patients, disturbances in body fat distribution do play a significant role (13). The body roundness index (BRI) has recently been considered a good estimate of fat distribution (14). In contrast to the traditional body mass index (BMI), the BRI better reflects the accumulation of visceral fat, taking into consideration the relationship between waist circumference and height. BRI is closely related to metabolic disorders, vascular stiffness, and increased inflammatory responses, further deteriorating the prognosis of stroke (15–17).

Although several single previous studies have discussed the value of SMI and BRI for stroke outcomes, few have explored the combination of both regarding the interaction between loss of muscle mass and fat distribution. This paper will analyze the data from patients with acute stroke and adopt multivariate analysis to confirm that SMI and BRI bear a combined predictive value for the unfavorable functional outcomes of stroke. In addition, we go further into the clinical value of their combination, leading to new perspectives and scientific evidence for the early identification and exact intervention of high-risk patients.

## Research methods

### Study design

This is a single-center, prospective cohort study that was used to determine the value of the SMI and BRI for the prediction of poor outcomes in patients with acute stroke. According to a predefined protocol, the primary exposures (SMI and BRI), the main outcome (90-day modified Rankin scale), and the core analyses (multivariable logistic regression and ROC) were specified before data collection. The study was approved by the Ethics Committee of Jiangwan Hospital, Hongkou District, Shanghai (No. 202111), and conducted in accordance with the Declaration of Helsinki.

### Study population

A total of 123 acute ischemic stroke patients admitted to the Jiangwan Hospital, Hongkou District, Shanghai from January 2022 to December 2023 were consecutively enrolled. Inclusion and exclusion criteria were as follows: Inclusion criteria: ① Age ≥18 years; ② Diagnosis of acute ischemic stroke confirmed by imaging (CT or MRI); ③ Admission within 3 days of stroke onset; ④ Capability to undergo lumbar imaging for accurate SMI measurement; ⑤ Signed informed consent and availability of complete baseline data, including age, sex, height, weight, waist circumference, education level, stroke subtype, NIHSS score, and pre-stroke mRS score. Exclusion criteria: ① Pre-stroke mRS score ≥2; ② Severe comorbidities such as recurrent malignancy (within the past 5 years), liver cirrhosis, or a life expectancy of less than six months; ③ Neuromuscular diseases (e.g., myositis, muscular dystrophy, neurodegenerative disorders); ④ Severe cardiovascular conditions (e.g., uncontrolled arrhythmia, heart failure, cardiac pacemaker implantation), peripheral arterial disease (e.g., lower limb artery stenosis or occlusion), or severe infections; ⑤ Uncontrolled metabolic or endocrine disorders (e.g., type 2 diabetes, thyroid disorders); ⑥ Chronic inflammation, autoimmune diseases, or immunodeficiency disorders (e.g., acquired immunodeficiency syndrome); ⑦ Inability to undergo imaging or complete follow-up.

### Sarcopenia diagnosis and grouping criteria

According to EWGSOP2 (2018) and AWGS (2019), sarcopenia requires low muscle mass together with low muscle strength ± impaired performance (12, 18). In our acute stroke cohort, standardized handgrip and performance tests were not feasible at admission due to hemiparesis; therefore, for analytic purposes, “sarcopenia” was operationally defined by low skeletal muscle mass (L3-SMI) using sex-specific thresholds referenced to these frameworks. Participants were categorized into the Sarcopenia group or non-sarcopenia group. This operational definition focuses on muscle quantity and does not represent a full diagnostic classification. The diagnostic domains and cutoff values according to the 2018 EWGSOP2 and 2019 AWGS consensus definitions are summarized in Supplementary Table S1.

### MRI assessment of skeletal muscle

Skeletal muscle was assessed using a 1.5T Siemens Avanto MRI system. The third lumbar vertebra (L3) midpoint was identified via axial cross-sectional imaging. Key measurements included: Definition of the L3 midpoint plane: The imaging slice showing the most prominent transverse processes of the L3 vertebra. Measurement of skeletal muscle cross-sectional area (SMA): Quantifying the cross-sectional area of skeletal muscle and fat tissue at the L3 plane to estimate lean body mass and fat content. Calculation of SMI: SMI was defined as the skeletal muscle area (cm^2^) at the L3 plane divided by height squared (m^2^). Thresholds for low SMI were ≤40.8 cm^2^/m^2^ for men and ≤34.9 cm^2^/m^2^for women. These cut-offs were originally derived from CT-based studies but have been commonly applied for comparability; our MRI measurements used the same L3 level and segmentation principles. To check robustness, SMI was also analyzed as a continuous variable, yielding consistent associations with outcomes.

### Anthropometric measurements

#### Body weight measurement

##### Ambulatory patients

Body weight was measured using an electronic scale placed on a flat, hard surface. Participants were measured in the morning, fasting, wearing light clothing, and without shoes. Readings were recorded to the nearest 0.1 kg after stabilization. Bedridden patients: Body weight was measured using a medical bed scale. The scale was zeroed before use, and the patient remained supine during the measurement.

#### Height measurement

##### Ambulatory patients

Height was measured using a stadiometer. Patients were asked to stand straight with heels together, toes separated by approximately 60 degrees, and body aligned against the stadiometer. The measurement was recorded to the nearest 0.1 cm. Bedridden patients: Height was estimated using the knee height method, measured from the lower edge of the knee to the sole of the foot. Formulas for calculating height were: Men: Height = 64.19 + (2.02 × knee height, cm); Women: Height = 84.88 + (1.83 × knee height, cm). Each knee-height measurement was taken twice by trained personnel, and the mean value was used to minimize random error.

#### Waist circumference measurement

##### Ambulatory patients

Measured using a flexible, inelastic tape measure (accuracy: 0.1 cm) at the midpoint between the anterior superior iliac spine and the lowest rib margin (narrowest waist point). Readings were taken at the end of exhalation. Bedridden patients: Waist circumference was measured in the supine position at the same anatomical landmarks.

#### Calculation of BRI

BRI was calculated using the formula proposed by Thomas et al. (14) $\mathrm{BRI}=364.2–365.5\times \sqrt{1-(\frac{\mathrm{WC}}{2\pi })÷{\left(0.5\times H\right)}^{2}}$ , where WC is waist circumference (m), and H is height (m).

### Outcome variables

#### Prognosis assessment

Functional outcomes were assessed 90 days post-discharge using the modified Rankin scale (mRS). Unfavorable outcomes were defined as an mRS score of 3–6, indicating moderate-to-severe disability or death.

#### Covariates

To adjust for potential confounders, baseline clinical characteristics, such as age, sex, smoking history, alcohol consumption, hypertension, diabetes, atrial fibrillation, and stroke severity (NIHSS score at admission), were included in the analysis.

### Statistical analysis

All analyses were performed using R software. Continuous variables were presented as mean ± standard deviation (SD), while categorical variables were reported as frequencies and percentages. Group comparisons used independent-sample *t*-tests for normally distributed continuous variables, Mann–Whitney *U* tests for non-normally distributed variables, and chi-square or Fisher’s exact tests for categorical variables. Multivariate logistic regression models were constructed to assess the independent predictive effects of SMI and BRI on unfavorable outcomes. Covariates were selected using stepwise regression to control for confounders, including age, sex, smoking, alcohol use, hypertension, diabetes, atrial fibrillation, and NIHSS score. Model performance was evaluated through: receiver operating characteristic (ROC) curves and calculation of the area under the curve (AUC), where AUC >0.8 indicates good predictive ability; calibration curves to assess consistency between predicted probabilities and observed outcomes; decision curve analysis (DCA) to evaluate the net clinical benefit and stability of the model across different probability thresholds. Some clinical factors potentially affecting prognosis-such as reperfusion treatment, dysphagia, nutritional status, and rehabilitation intensity-were not systematically collected and therefore were not included in the regression model. Likewise, data on medication use (e.g., statins, antihypertensive, antidiabetic, and corticosteroid therapies) were not consistently available and thus were not incorporated as covariates.

## Results

### Baseline characteristics of patients

The baseline characteristics of the patients are presented in Table 1. The sarcopenia group had a significantly lower SMI compared to the non-sarcopenia group (*p* < 0.001). However, no statistically significant differences were observed between the two groups in terms of age (*p* = 0.318), sex distribution (*p* = 0.887), systolic blood pressure (*p* = 0.474), diastolic blood pressure (*p* = 0.333), pulse (*p* = 0.654), and BRI (*p* = 0.213). In terms of lifestyle and comorbidities, the two groups did not differ significantly in smoking history (*p* = 0.106), diabetes mellitus (*p* = 0.061), alcohol consumption (*p* = 0.662), hypertension (*p* = 0.785), atrial fibrillation (*p* = 0.915), and previous stroke history (*p* = 0.741). The distribution of TOAST stroke subtypes also showed no statistically significant differences between the two groups (*p* > 0.05).

**Table 1:** Baseline characteristics of non-sarcopenia and sarcopenia groups.

Variable	Non-sarcopenia (*N* = 80)	Sarcopenia (*N* = 43)	*p*-value
SMI (cm^2^/m^2^)	47.484 ± 5.934	33.722 ± 3.307	<0.001
Age (years)	68.875 ± 12.464	71.279 ± 13.054	0.318
Gender			0.887
Female	42 (52.500%)	22 (51.163%)	
Male	38 (47.500%)	21 (48.837%)	
SBP (mmHg)	146.800 ± 20.037	144.116 ± 19.177	0.474
DBP (mmHg)	84.350 ± 12.763	82.186 ± 9.634	0.333
Pulse (bpm)	79.287 ± 11.157	78.256 ± 13.831	0.654
BRI	3.787 ± 0.936	4.067 ± 1.545	0.213
NIHSS score (1d)	4.800 ± 5.718	4.140 ± 6.186	0.554
Alcohol (%)			0.662
Yes	9 (11.250%)	6 (13.953%)	
No	71 (88.750%)	37 (86.047%)	
Smoking (%)			0.106
Yes	19 (23.750%)	5 (11.628%)	
No	61 (76.250%)	38 (88.372%)	
Hypertension (%)			0.785
Yes	28 (35.000%)	14 (32.558%)	
No	52 (65.000%)	29 (67.442%)	
Diabetes (%)			0.061
Yes	22 (27.500%)	19 (44.186%)	
No	58 (72.500%)	24 (55.814%)	
Atrial fibrillation (%)			0.915
Yes	6 (7.500%)	3 (6.977%)	
No	74 (92.500%)	40 (93.023%)	
Stroke (%)			0.741
Yes	13 (16.250%)	8 (18.605%)	
No	67 (83.750%)	35 (81.395%)	
TOAST classification			0.671
LAA	30 (37.500%)	21 (48.837%)	
CE	13 (16.250%)	6 (13.953%)	
SVO	30 (37.500%)	14 (32.558%)	
OC	2 (2.500%)	0 (0.000%)	
UE	5 (6.250%)	2 (4.651%)	

### Univariate binary logistic regression analysis

**Figure 1:**
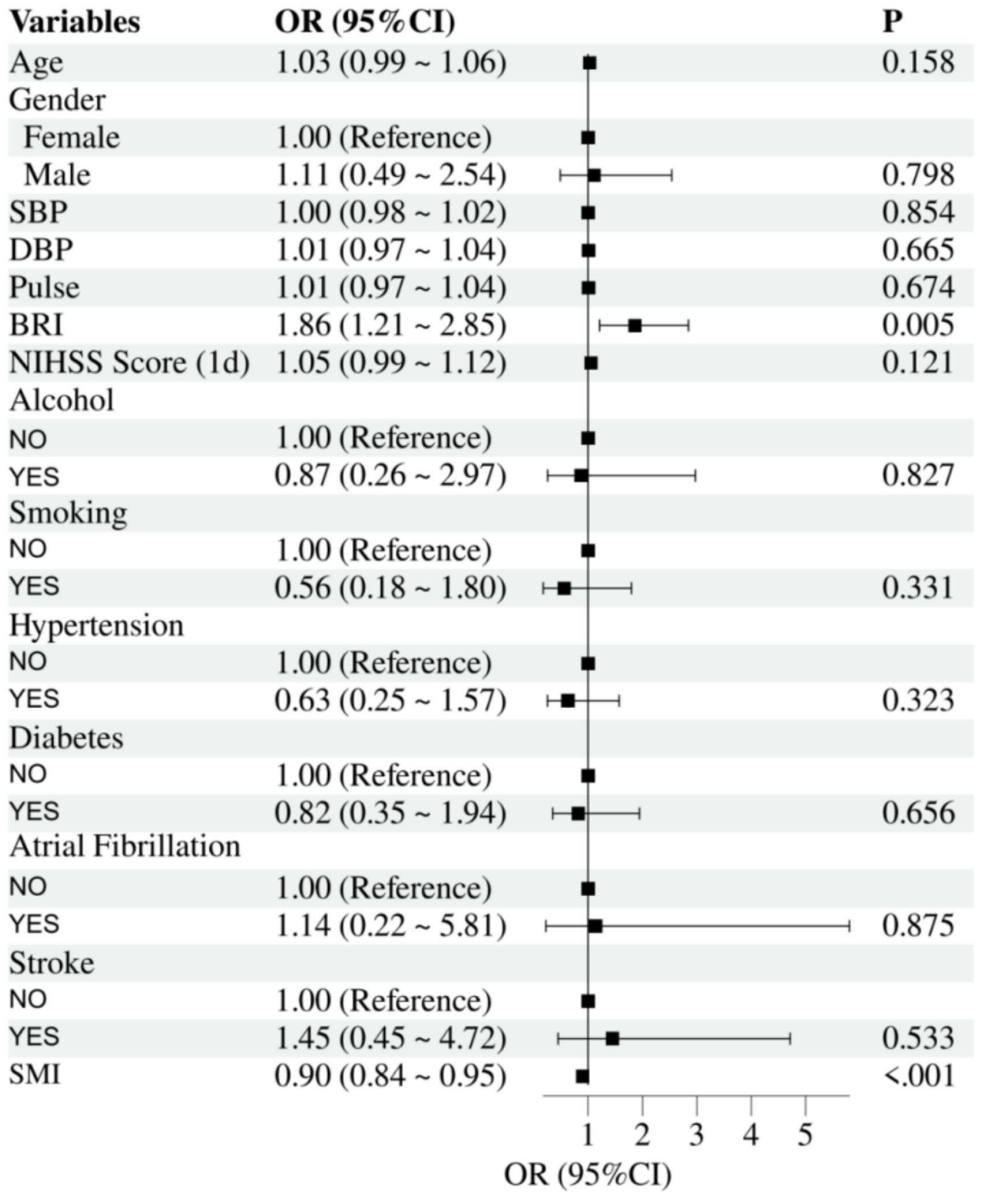
Univariate forest plot of binary logistic regression for prognostic outcomes.

The results of the univariate binary logistic regression analysis are presented in Figure 1. The analysis demonstrated that the SMI significantly influences unfavorable outcomes in acute stroke patients. The odds ratio (OR) for SMI was 0.90 (95% CI, 0.84–0.95, *p* < 0.001), indicating that for every unit increase in SMI, the risk of unfavorable outcomes decreases by 10%. Similarly, the BRI was significantly associated with an increased risk of unfavorable outcomes, with an OR of 1.86 (95% CI, 1.21–2.85, *p* = 0.005), suggesting that higher BRI values are strongly correlated with worse prognosis. Other covariates, including age, sex, systolic blood pressure, diastolic blood pressure, pulse, NIHSS score, history of alcohol consumption, smoking, hypertension, diabetes, atrial fibrillation, and prior stroke, did not reach statistical significance (*p* > 0.05).

### Multivariate regression analysis

Results of multivariable regression analyses for the association of sarcopenia and BRI with adverse outcomes among acute stroke patients in Table 2. Results from model adjust I adjusted only for age and sex, demonstrated that sarcopenia resulted in an OR of 11.726 (95% CI, 4.380–31.394, *p* < 0.001), indicating the risk of adverse outcomes in sarcopenia patients was 11.7-fold higher than those without sarcopenia. At the same time, BRI also represented a significant association with an odds ratio of 1.814 (95% CI, 1.172–2.807, *p* = 0.008), representing a worse prognosis owing to abnormal fat distribution. Afterward, model adjust II, with further adjustment for more covariates including alcohol consumption, smoking, hypertension, diabetes, previous stroke, atrial fibrillation, and NIHSS score, the OR for sarcopenia remarkably increased to 33.470 (95% CI, 7.118–157.394, *p* < 0.001), indicating that sarcopenia remained a powerful and independent predictor of unfavorable outcome even after adjustment for several confounders. The BRI also yielded an even higher OR of 2.200 (95% CI, 1.212–3.992, *p* = 0.010), again confirming that abnormal fat distribution enhances the risk significantly. The associations between SMI and unfavorable outcomes remained directionally consistent when SMI was treated as a continuous variable, confirming the stability of the results.

**Table 2:** Multiple regression analysis for the association between sarcopenia and BRI with outcome.

Exposure	Adjust I	Adjust II
Non-sarcopenia	Reference	Reference
Sarcopenia	11.726 (4.380, 31.394) < 0.00001	33.470 (7.118, 157.394) < 0.00001
BRI	1.814 (1.172, 2.807) 0.00753	2.200 (1.212, 3.992) 0.00950

Adjust I model: Adjusted for gender and age. Adjust II model: Adjusted for gender, age, alcohol, smoking, hypertension, diabetes, stroke, atrial fibrillation, and NIHSS score.

### ROC curve analysis

**Figure 2:**
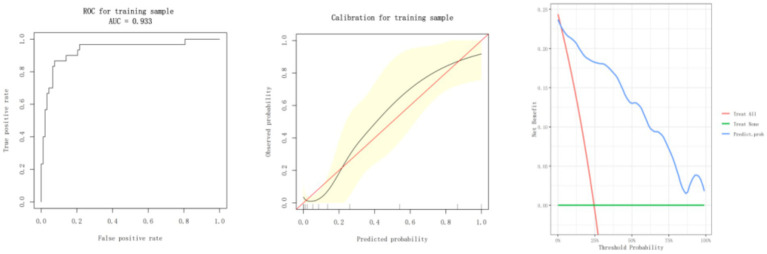
Model performance evaluation with ROC curve, calibration curve, and decision curve analysis.

Figure 2 shows the performance of the predictive model in terms of the ROC curve, calibration plot, and DCA. ROC analysis revealed the model’s robust discriminative capability, achieving an area under the AUC of 0.933. This suggests a very good discriminative ability for poor prognosis in acute stroke patients. The calibration curve showed the actual probabilities of patients’ outcomes in the model, and the observed probabilities were closely related. The black prediction curve is very close to the ideal diagonal red line, which also means that the above model is reliable. The decision curve analysis confirmed that for all probability thresholds, the model yields a higher net clinical benefit than a “treat all” or “treat none” approach.

## Discussion

This study evaluated the combined predictive ability of SMI and BRI for unfavorable outcomes in acute stroke patients. The findings demonstrate that low SMI and high BRI are independent risk factors for poor outcomes, and their combination significantly improves the discriminative ability of the predictive model (AUC = 0.933). These results provide important insights for the early identification and personalized intervention of high-risk patients in clinical practice.

Patients with diminished SMI demonstrated a significantly elevated risk of unfavorable outcomes (OR = 33.470, *p* < 0.001), consistent with previous studies. For example, research by Honma et al. (19) demonstrated that stroke patients with low SMI face higher mortality during the rehabilitation period and experience delayed functional recovery. In this study, the mean SMI in the sarcopenia group was 33.722 cm^2^/m^2^, significantly lower than the 47.484 cm^2^/m^2^ observed in the non-sarcopenia group (*p* < 0.001). This underscores the critical role of skeletal muscle in stroke recovery (20). Skeletal muscle serves as a key metabolic organ, directly influencing mobility and nutritional status (21). To our knowledge, this is the first study to validate the prognostic utility of SMI in acute stroke patients, suggesting that rapid SMI assessment during admission could aid in prognosis evaluation and treatment planning. The imaging-based assessment of skeletal muscle at the L3 level is highly objective and reproducible, enhancing its clinical applicability.

The study also identified BRI as an important independent predictor, with elevated BRI significantly increasing the risk of poor outcomes (OR = 2.200, *p* = 0.010). Compared to traditional BMI, BRI more accurately reflects abdominal fat distribution, making it particularly useful for evaluating metabolic status (22). Previous studies, such as those by Liu et al. (23), have linked high BRI with increased risks of stroke recurrence and mortality. Abdominal fat accumulation may worsen prognosis by exacerbating metabolic dysregulation and endothelial dysfunction. The simple calculation of BRI using waist circumference and height makes it a practical tool for rapid application in resource-limited clinical settings (24–26).

Furthermore, our findings support the growing recognition that skeletal muscle and visceral fat are not isolated factors, but part of an interconnected metabolic network (27). Sarcopenia is often associated with chronic low-grade inflammation, insulin resistance, and oxidative stress, while visceral adiposity contributes to these processes through the secretion of pro-inflammatory adipokines such as IL-6 and TNF-α (28). Low SMI may reduce metabolic reserve and impair the secretion of protective myokines (e.g., irisin and IL-10), leading to weakened anti-inflammatory capacity and muscle regeneration, whereas high BRI reflects visceral fat accumulation that promotes systemic inflammation, endothelial dysfunction, and pro-thrombotic states. These converging mechanisms can aggravate neurological injury and increase mortality or poor functional recovery after stroke. These shared pathways may help explain the amplified risk when both low SMI and high BRI are present.

Recent studies have suggested that the combination of muscle mass and fat indicators—such as the muscle-to-fat ratio or sarcopenic obesity index—provides a more accurate risk profile in elderly and post-stroke populations (29, 30). Our results align with these perspectives and further extend them to functional stroke outcomes.

The novelty of this study lies in the integration of SMI and BRI for predicting unfavorable functional outcomes in acute stroke patients. It had an AUC of 0.933, strikingly superior to those based on either single metric. The ROC curves and AUC values were calculated using the pROC package in R, with 95% confidence intervals estimated by the DeLong method and optimal thresholds identified by Youden’s index. Skeletal muscle loss and abnormal fat distribution may be two independent factors affecting the prognosis of stroke, probably through certain complementarities. The decline in recovery ability due to insufficient SMI may be further aggravated by the increase in fat; metabolic disorders that accompany high BRI inhibit muscle synthesis. Such a vicious circle has been established herein (31). The multivariable regression analysis and decision curve analysis further demonstrated the new combined model with robustness and good clinical utility. It adds a new model not only for the stratified management of the patients but also opens up a new horizon for the prognostic study of other diseases. Compared with the existing studies, it further advanced the analytical depth and clinical applicability. For instance, Abe et al. (20) indicated that when SMI alone was used to predict stroke outcomes, the AUC was merely 0.682. In the current study, the incorporation of BRI significantly improved its performance. The calibration curves showed good agreement between the predicted probabilities and the observed outcomes, while the decision curve analysis showed a consistent net benefit at different thresholds, indicating the clinical relevance of the combined model.

Clinical practice of the model will enable rapid identification of high-risk patients in the acute phase of stroke by imaging and basic measurements for the delivery of SMI and BRI. These insights offer a scientific rationale for designing personalized intervention strategies.

Some limitations of the present study should be underscored despite its strengths. In particular, strength and performance were not systematically assessed at admission. Thus, we used low SMI as an operational exposure rather than a full EWGSOP2/AWGS sarcopenia diagnosis. In addition, because lumbar MRI at the L3 level was required for inclusion, a few otherwise-eligible patients could not complete scanning due to clinical instability or contraindications (e.g., pacemaker, severe agitation), which may introduce potential selection bias compared with standard head-only imaging workflows. The single-center design and relatively small sample size of this study may limit its generalizability to broader populations. In addition, because imaging assessment requires technical expertise, it most probably will restrain the SMI measurement in some way. Moreover, as highlighted in a recent study, the lack of standardized imaging protocols and analysis methods for skeletal muscle across centers may further affect the reproducibility and clinical implementation of SMI-based assessments (32). Large sample sizes are needed in future studies to establish the validity of the model by prospective multicenter research. Furthermore, future research may benefit from incorporating dynamic monitoring of muscle-fat parameters, combining radiomics and artificial intelligence techniques to improve prediction accuracy and bedside usability. Dynamic metrics, arc circumference, and muscle mass have the potential to provide long-term prognostic monitoring. Moreover, certain potential confounders such as reperfusion therapy, swallowing impairment, nutritional deficiency, and rehabilitation intensity were not available in this dataset, which might have influenced functional recovery to some extent. However, the main associations of SMI and BRI with unfavorable outcomes remained consistent after multivariate adjustment for key demographic and clinical variables. In addition, medication effects could not be fully accounted for, as baseline data on statin, antihypertensive, antidiabetic, and corticosteroid use were incomplete. Nevertheless, the associations of SMI and BRI with functional outcomes were robust across adjusted models, suggesting that these unmeasured factors are unlikely to substantially alter the main conclusions.

In summary, our study shows that combining SMI and BRI provides a more accurate prediction model for identifying stroke patients at risk of poor functional outcomes. The strong predictive power of the combined model (AUC = 0.933) suggests a potential complementary effect of muscle wasting and visceral fat in influencing stroke recovery. As both indicators are accessible and objective, they may be applied in early clinical screening and individualized rehabilitation planning. Future large-scale, multicenter studies are needed to validate these findings and assess their utility in different clinical settings.

## Data Availability

The original contributions presented in the study are included in the article/Supplementary material, further inquiries can be directed to the corresponding author.
