# Supplementary material for: Integrating skeletal muscle index and body roundness index for predicting functional outcomes in acute stroke patients: a prospective observational study
Source: Front Neurol. 2025 Oct 30;16:1643247. doi: 10.3389/fneur.2025.1643247 (PMC12611662; doi:10.3389/fneur.2025.1643247)
Supplement: Supplementary file 1 [file Table_1.docx]

**Table S1. Diagnostic domains and cutoff values for sarcopenia according to EWGSOP2 (2018) and AWGS (2019) consensus definitions**

| **Domain** | **EWGSOP2 (2018)**  **European Working Group on Sarcopenia in Older People 2** | **Cut-off (Male / Female)** | **AWGS (2019)**  **Asian Working Group for Sarcopenia** | **Cut-off (Male / Female)** |
| --- | --- | --- | --- | --- |
| Muscle Strength | Handgrip strength or chair stand test as entry criterion | <27 kg / <16 kg (handgrip)  >15 s (chair stand) | Handgrip strength or chair stand test as screening tool | <28 kg / <18 kg (handgrip)  ≥12 s (chair stand) |
| Muscle Quantity | Low muscle mass confirmed by DXA, BIA, CT or MRI | DXA ASM/height² <7.0 / <5.5 kg/m² | Low muscle mass measured by DXA or BIA | DXA ASM/height² <7.0 / <5.4 kg/m²  BIA ASM/height² <7.0 / <5.7 kg/m² |
| Physical Performance | Low performance indicates severe sarcopenia | Gait speed <0.8 m/s  SPPB ≤8 | Used for diagnosis and severity classification | Gait speed <1.0 m/s  SPPB ≤9 |
| Diagnostic Pathway | Low strength → confirm low mass → evaluate performance | - | Possible sarcopenia → confirm with muscle mass and/or performance | - |
